# Ameliorative effect of pomegranate peel extract nanoparticles and ovarian stem cells-derived exosomes on cyclophosphamide-induced premature ovarian insufficiency

**DOI:** 10.1186/s40659-025-00664-9

**Published:** 2026-01-20

**Authors:** Hamdy Y. Ismail, Mariam F. Farid, Nora A. Shaker, Tahsin Shoala, Adel F. Tohamy, Marwa A. Ibrahim, Hamdy Rizk, Y. R. Wally

**Affiliations:** 1 Department of Anatomy and Embryology, Faculty of Veterinary Medicine, Cairo University, Giza, Egypt; 2 Department of Environmental Biotechnology, College of Biotechnology, Misr University for Science and Technology, Giza, Egypt; 3 Department of Toxicology & Forensic Medicine, Faculty of Veterinary Medicine, Cairo University, Giza, Egypt; 4 The Bahamas Agriculture and Marine Science Institute, North Andros, The Bahamas; 5 Department of Biochemistry and Molecular Biology, Faculty of Veterinary Medicine, Cairo University, Giza, Egypt

**Keywords:** Fertility, Cyclophosphamide, Nanotechnology, Exosome, *Punica granatum*

## Abstract

**Background:**

Premature ovarian insufficiency (POI) is a fertility disorder impacting women under 40, characterized by an early deterioration of ovarian function, and is one of the major side effects caused by chemotherapy. Cyclophosphamide is a powerful chemotherapeutic agent used in various cancers; however, it inflicts substantial harm on other tissues, particularly the gonads, leading to temporary or permanent infertility.

**Materials and methods:**

Forty female albino rats *(Rattus norvegicus)* were divided into four groups. Group I (control group) received normal saline, then premature ovarian insufficiency was induced in the remaining groups by intraperitoneal injections of cyclophosphamide (CLP). After that, Group II received no treatment. Group III was administered a daily oral dose of pomegranate (*Punica granatum*) nanoparticles for one month. Group IV received a daily oral dose of pomegranates, as did group III, plus ovarian stem cell-derived exosomes via intraperitoneal injection twice weekly for one month. Rats were euthanized 30 days post-POI induction; blood was then collected to evaluate hormone levels, and sections of the ovaries were collected for histopathological analysis. Frozen sections were procured for gene expression and oxidative stress studies.

**Results:**

The hormonal assessment of groups indicated a notable reduction in estrogen (E2) level and an elevation of follicle-stimulating hormone (FSH) in group II compared to the control and treated groups. Additionally, the ovaries of group II exhibited pronounced degeneration of ovarian follicles, accompanied by the desquamation of granulosa cells. Gene expression study indicated a downregulation of FSHR, CYP19A1, and AMH in the same group. Rats in both groups III and IV exhibited an increased number of follicles, improved ovarian shape, a considerable elevation in blood E2, a marked decrease in serum FSH levels, and an up-regulation of the three examined genes.

**Aim of work:**

The study aimed to assess the therapeutic efficacy of pomegranate (*Punica granatum*) peel extract nanoparticles alone and their synergistic effect with ovarian stem cell exosomes in reversing premature ovarian insufficiency (POI) caused by cyclophosphamide.

**Conclusion:**

Treatment with (*Punica granatum*) nanoparticles and exosomes partially enhanced the structure and function of the ovaries, thereby alleviating the adverse effects of Cyclophosphamide.

## Introduction

 Premature ovarian insufficiency (POI) is a significant long-term adverse effect among young cancer patients. Up to 1% of women suffer from POI, which is characterized by the loss of ovarian function before the age of 40. POI has similar side effects to menopause, such as infertility, hot flashes, osteoporosis, and sexual dysfunction [1]. Chemotherapy, autoimmune disorders, congenital anomalies, or endocrine problems can cause this condition. Among cancer therapies, cyclophosphamide is a chemotherapeutic drug frequently used in the treatment of various malignancies. Its primary objective is to eliminate rapidly dividing cancerous cells by inducing DNA damage and initiating apoptotic pathways through the release of *cytochrome c* from the mitochondria. Conversely, cyclophosphamide also affects cells with naturally high turnover rates, such as ovarian granulosa cells (GCs), which are essential for follicle formation and oocyte survival, ultimately leading to POI [2–5].

Multiple approaches have been used to address POI. The first-line treatment has been hormone replacement therapy (HRT), followed by more innovative therapies such as exosomes, stem cell therapy, and the use of nanotechnology [6]. While conventional treatments address POI symptoms, there remains a need for safer, more effective interventions that preserve ovarian function and fertility with fewer risks. Stem cells utilize a paracrine signaling system to release exosomes, which, in turn, participate in intercellular communication and transport cytokines, microRNAs (miRNAs), long non-coding RNAs (lncRNAs), and small molecular proteins to recipient cells to regulate their function [6]. Also, nanotechnology involves the design and synthesis of nanoparticles with sizes ranging from 1 to 100 nm. Their small size and large surface area enable them to interact with tissues and cells at subcellular levels [7]. The intersection of stem cell–derived paracrine signalling, exosome-mediated communication, and nanotechnology offers a promising approach to enhance ovarian repair while potentially minimizing related hazards. However, gaps persist regarding optimal sources of exosomes, their concentration, mechanisms of exosomes in ovarian restoration, and the most suitable nanoparticle systems to maximize targeted delivery, safety, and efficacy in POI patients.

This study aims to support current evidence on regenerative strategies for POI, including the use of stem cells and their derived exosomes. Furthermore, evaluate the therapeutic effect of ovarian stem cell-derived exosomes to support granulosa cell survival and folliculogenesis. Finally, explore the therapeutic potential of *Punica granatum-derived* phenolics to enhance antioxidative pathways relevant to POI. By integrating insights from stem cell biology, nanotechnology, and phytochemical research, the work aims to identify safer approaches to preserve ovarian reserve and improve the quality of life for young women facing POI.

## Materials and methods

### Study protocol

The Institutional Animal Care and Use Committee of Cairo University’s Veterinary Medicine (Vet-94 CU-IACUC) reviewed and approved the study protocol, approval number (Vet CU13102024973).

### Animals

Forty mature female white albino rats (*Rattus norvegicus)* weighing 250–300 g were maintained at controlled temperatures of 23 ± 2 °C and light conditions of 14 h of illumination and 10 h of darkness for 1 week to acclimate with ad libitum access to rat chow and water.

### Animal grouping

The rats were divided into four groups (*n* = 10). Group I (control negative) received daily oral normal saline for one month; groups II, III, and IV were induced to develop POI by intraperitoneal administration of cyclophosphamide (50 mg/kg) followed by 8 mg/kg for 15 consecutive days [8–12]. Then, Group II (control positive or CLP group) received no treatment. Group III (*Punica granatum* treated) received a daily oral dose of pomegranate nanoparticles (1.47 mg/kg) for one month [13, 14]. Group IV (pomegranate nanoparticles + ovarian stem cell-derived exosomes) received a daily dose of pomegranates, as in group III, combined with a volume of 100 µL of exosomes (1 ml Phosphate-buffered saline (PBS) containing 1 × 10^10^ particles of ovarian stem cell-derived Exosomes) via intraperitoneal injection twice weekly for a month [15].

### Preparation of *Punica granatum* nano-formulation

The pomegranate nanoparticles were created utilizing the Hamouda et al. [16] approach with minor changes [17, 18]. Pomegranate solution (14% v/v), ethanol (3% v/v), and biosurfactant non-ionic Tween 80 (3% v/v), which accounted for 20% (v/v) of the total emulsion, were combined to create the nano-emulsions. The resultant emulsion was mixed and heated to 86 °C for one hour, and then the mixture was mixed with 80% water and allowed to sit at room temperature (25 ± 3 °C) for three minutes before being centrifuged at 10,000 × g using an LMC-4200R Laboratory Refrigerated Centrifuge, Heliopolis, Cairo 11,757, Egypt.

### Characterization of *Punica granatum* nanoparticles

- Transmission electron microscope

The pomegranate nanoparticles were reported using electron microscopy. Using a JEOL JEM-1010 transmission electron microscope (Peabody, MA 01960, USA) adjusted at 70 kV, electron micrographs were collected at Azhar University’s Regional Centre for Mycology and Biotechnology (RCMB) [19].

- Zeta potential

Using a Zeta Plus tool (Malvern Zetasizer Nano-zs90, Malvern Instruments Ltd.), photon correlation spectroscopy was used to examine the zeta potential [18].

- Fourier transform infrared spectroscopy (FTIR)

The pomegranate sample was dried, and powdered pomegranate extract was directly placed on the diamond ATR crystal. Pressure was applied to ensure good contact, and a standard FTIR spectrometer with an attenuated total reflection (ATR) attachment was used [20].

- X-Ray diffraction (XRD)

The pomegranate sample was finely ground and packed into a sample holder, and the surface was flattened. A powder X-ray diffractometer with Cu-Kα radiation (λ = 1.5418 Å) was used [21].

### Isolation of ovarian stem cell-derived exosomes

Exosomes were extracted from the culture supernatants of ovarian stem cells (OSCs). The medium of the cells was changed to serum-free medium once the cells had achieved 80% confluence, and they were then cultured for 48 h. The sample is then put through a series of centrifugation procedures. Centrifugation is performed on the sample for 10 min at 500 × g to remove cells, 20 min at 2000 × g to remove cell debris and apoptotic bodies, 30 min at 10,000 × g to separate large micro vesicles, and finally, 70 min at 100,000 × g to pellet the exosomes. To separate the exosome, the pellet is washed with PBS and then put through one more round of ultracentrifugation [22, 23].

### Characterization of OSC-derived exosomes

- Transmission electron microscope examination

A drop of the exosome solution was applied to the carbon-coated copper grids (CCG) that had been stained with uranyl acetate solution (2% w/v) as a negative stain for four minutes. The grids were then allowed to air dry at room temperature after any excess liquid was removed using filter paper. The JEOL JEM-1010 transmission electron microscope was then used to create electron micrographs at the Regional Center for Mycology and Biotechnology (RCMB) at Al-Azhar University [24].

- Iron oxide nanoparticle labeling for exosomes

In a humid CO_2_ incubator, 200 mg/ml of iron oxide nanoparticles were added to the growing OSCs in DMEM for a full day. After 48 h, the culture media was changed. Exosomes with labels were isolated via ultracentrifugation. The animals in the positive control group received iron-loaded exosomes. The migration of exosomes to ovarian tissue was validated by iron nanoparticles discovered in ovarian tissue [23].

### Vaginal smears

After identifying and distributing the female rats, a vaginal smear from each group was prepared on a glass slide. This procedure was repeated following induction in groups II, III, and IV, as well as after treatment in groups III and IV. A cotton-tipped swab wet with regular saline was carefully inserted into the rats’ vaginal cavities to get the smear. Before being removed, the swabs were delicately rolled around and softly placed against the vaginal wall. As soon as the moist swab was removed from the vaginal cavity, it was rolled or smeared onto a clean glass slide. The interval between the collection of smears was 4–12 h [25].

### Hormonal analysis

At the end of the trial, serum FSH and E2 levels were measured using ELISA kits (SinoGeneClon Biotech Co.) to assess ovarian function. Blood was drawn from all groups’ retroorbital veins in the morning, and the serum was separated and stored at − 20 °C until analysis [26].

### Histopathology

- H&E examination

The specimens were put in 10% neutral buffered formalin for fixation; then, the samples were cut, cleaned with water, dehydrated in increasing ethyl alcohol grades, clarified in xylene, and embedded in paraffin. Hematoxylin and eosin stain was used to prepare and stain thin Sections (4–6 µ) [27].

- Morphometric study

Sections under a light microscope were photographed on the screen using a fixed video camera. Antral follicle counts were taken from H&E sections of several experimental groups [28].

- Immunohistochemistry staining protocol

Paraffin sections were mounted on positively charged slides by using the avidin-biotin-peroxidase complex (ABC) method. Sections from each group were incubated with these primary antibodies (Table 1), then the reagents required for the ABC method were added (Vectastain ABC-HRP kit, Vector Laboratories). Marker expression was labeled with peroxidase and colored with diaminobenzidine (DAB, produced by Sigma) to detect the antigen-antibody complex. Negative controls were included using non-immune serum in place of the primary or secondary antibodies. IHC-stained sections were examined using an Olympus microscope (BX-63).

**Table 1:** Antibody set used in immunohistochemistry of the current study

Antibody	Company name	Type	Host	Catalogue No.	Dilution
Caspase 3	ABclonal	Polyclonal	Rabbit	A11953	1:100
PCNA	ABclonal	Polyclonal	Rabbit	A13336	1:100

### Quantitative reverse transcription-polymerase chain reaction (qRT-PCR)

RNA was extracted following the manufacturer’s guidelines using the Qiagen RNeasy mini-Kit (cat no. 74004). For cDNA synthesis, the RevertAid First Strand cDNA Synthesis Kit from Thermo Scientific (cat. no. K1621) was utilized [29]. Gene expression was quantified using the SYBR Green master mix and the StepOnePlus™ Real-Time PCR System (Thermo Fisher Scientific, US) [30]. A list of gene-specific primers was used (Table 2).The PCR cycling conditions included 40 cycles with denaturation at 95 °C for 30 s, annealing at 58 °C for 30 s, and extension at 72 °C for 30 s [31]. Melting curve analysis was performed to confirm specificity. The relative quantification of gene expression was calculated using the ΔΔCt method. β-actin, a housekeeping gene, was used to normalize the gene expression data [29]. Each experiment included no template controls for all genes, and all samples were duplicated [32].

**Table 2:** Primer sets of assessed genes in the current study

	Sense	Antisens	Ampli-con	Accession no.
*FSHR*	TCATCCGCTACCCACAACTT	TGCACACACCCAGAGAGATT	233	NM_199237.2
*CYP19A1*	TGACGTCACTGACAACTCGG	CAAGTCCACGACAGGCTGAT	235	NM_017085.2
*AMH*	CTGTTTGGCTCTGATTCCCG	GTCTCTAGGAAGGGGTCAGC	179	NM_012902.2

### Oxidative stress marker assessment

Reduced glutathione (GSH) and malondialdehyde (MDA) products were identified as oxidative stress marker indicators by Beutler et al. (1963) and Ohkawa et al. (1979), respectively. Before being dissected, the ovarian tissues were briefly perfused with a phosphate-buffered saline solution. They were then homogenized using a tissue homogenizer in 5 milliliters of cold buffer (50 milligrams of potassium phosphate, pH 7.5) per gram of tissue. After centrifugation (4000 rpm for 15 min at 4 °C), the supernatant was extracted and utilized following the kit’s instructions for the malondialdehyde and glutathione-reduced assay.

### Statistical analysis

The data was analyzed using OriginPro, version 2016, a one-way ANOVA was conducted, and the effects of the various therapies were compared utilizing the post hoc test when *P* ≤ 0.05. The superscript letters represented the groups’ substantial differences.

## Results

### Confirmation of POI by vaginal smears

**Fig. 1:**
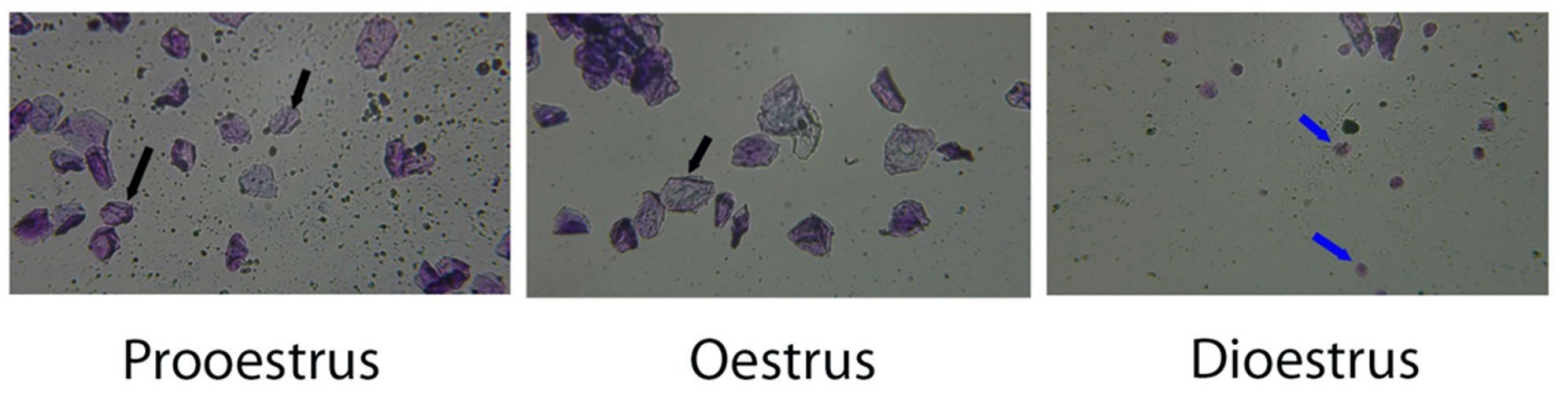
Stages of the estrous cycle in rats. A nucleated epithelial cell (black arrows), and neutrophils (blue arrows) (×400)

Rats in each group had their estrous cycles monitored for two weeks. The rats in the control group displayed a typical estrous cycle encompassing diestrus (59 h), metestrus (5 h), proestrus (18 h), and estrus (25 h), as shown in Fig. 1. Rats in the untreated POI group had erratic cycles, with some showing no regularity and others having a longer diestrus and a shorter estrous phase, and only 10% of the rats in the group had a typical estrous cycle. The findings demonstrated that the POI model was successfully generated, and that rats’ ovarian function was reduced following cyclophosphamide injection. Rats’ ovarian function was considerably enhanced, and their typical estrous cycle lengthened following treatment with pomegranate nanoparticles and their combination with OSC-derived exosomes.

### Characterization of pomegranate peel extract nanoparticles

- Transmission electron microscope (TEM) examination of nanoparticles

**Fig. 2:**
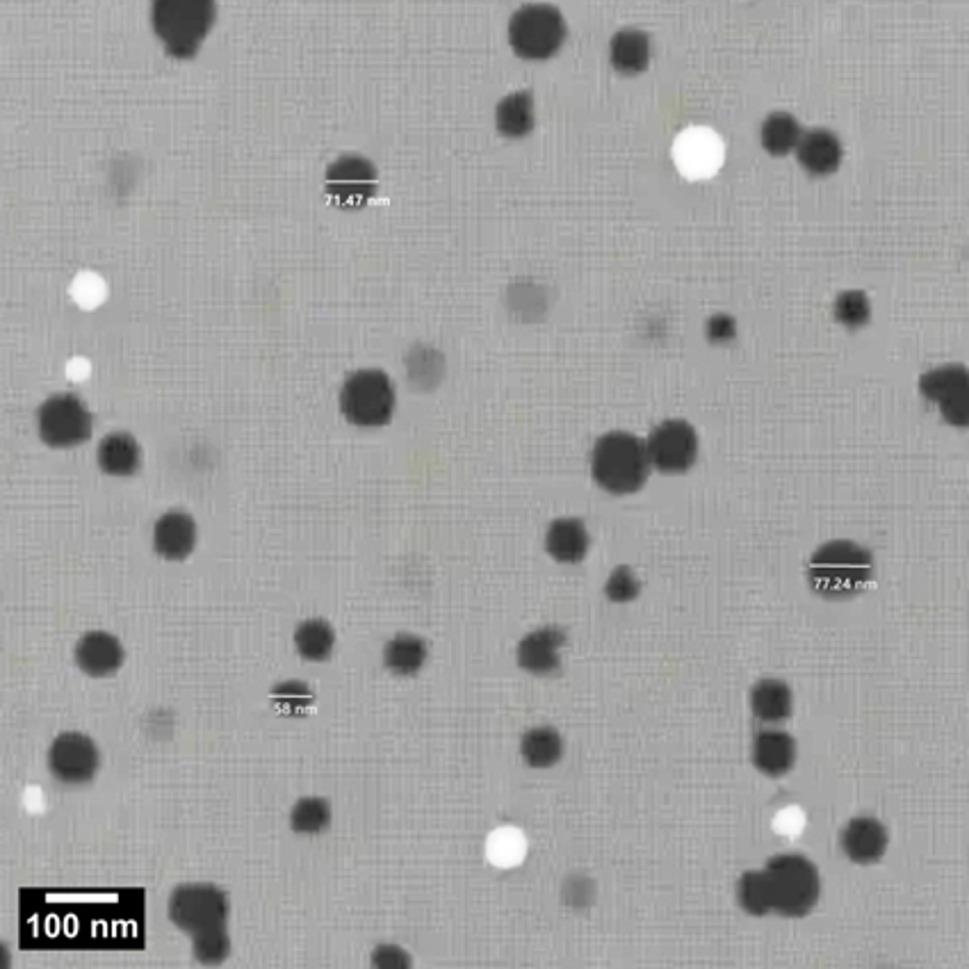
Transmission electron microscope image of prepared pomegranate nanoparticles, direct magnification 6000×, scale bar 100 nm

The TEM findings showed that the pomegranate nano-emulsion was irregularly spherical, with an average size of 69 nm. (Fig. 2)

- Zeta potential

**Fig. 3:**
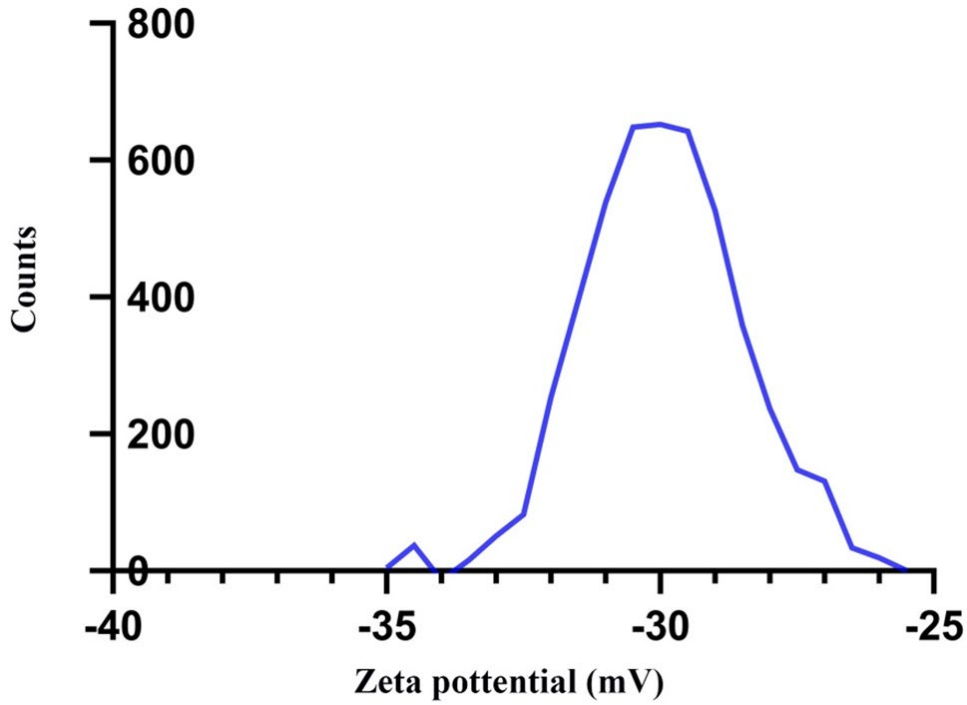
Zeta potential of pomegranate peel extract nanoparticles

The zeta potential indicated elevated values for charged particles, inhibiting aggregation and facilitating redispersion through repulsive electric forces, thus indicating good stability of the nanoparticles. (Fig. 3**)**

- Fourier transform infrared spectroscopy (FTIR)

**Fig. 4:**
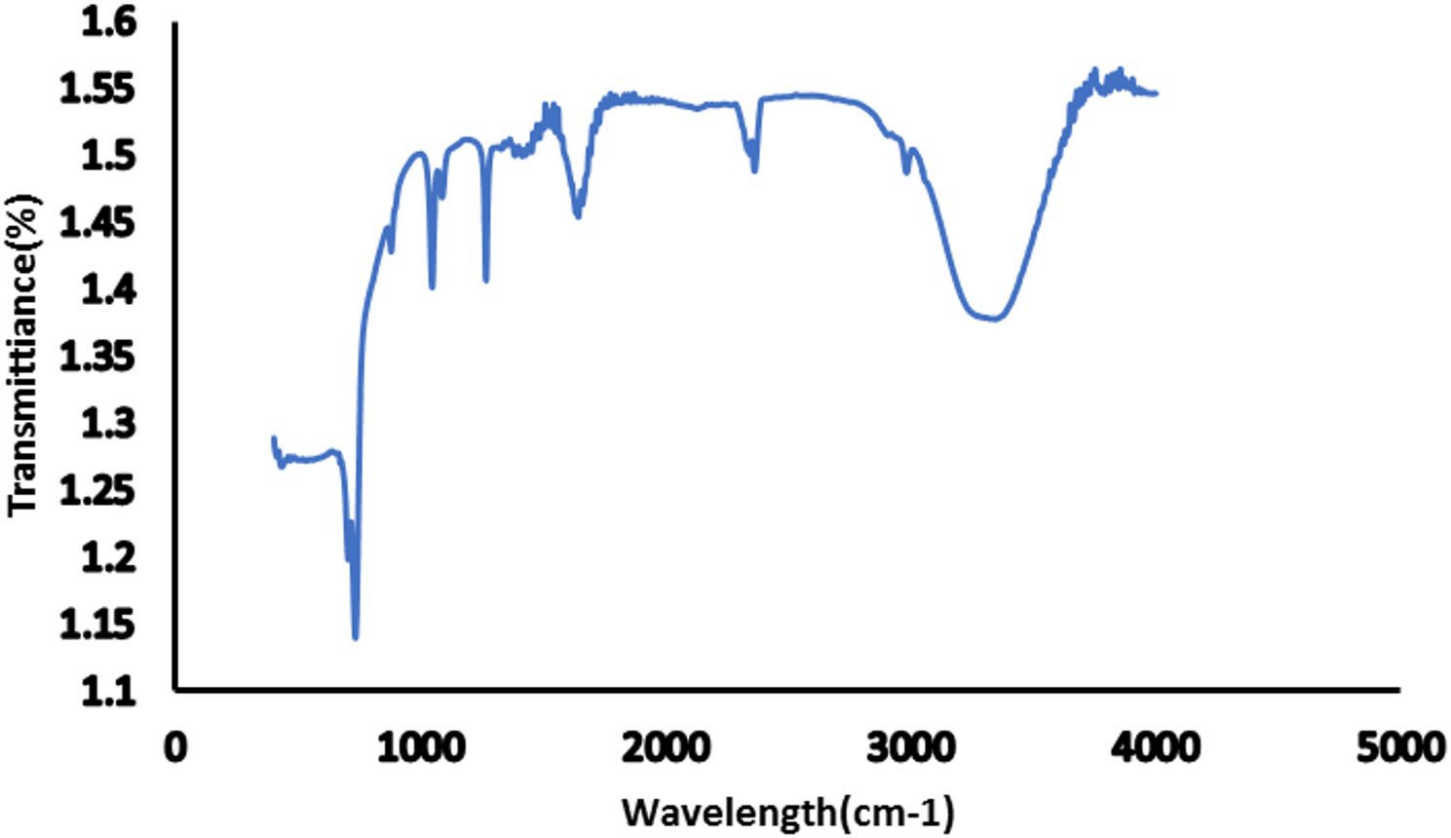
Fourier transform infrared spectroscopy of pomegranate peel extract nanoparticles showing absorption bands of different groups. The prominent peak at 3566 CM^− 1^ indicates the presence of phenolic compounds that contribute to the antioxidant activity

The FTIR spectrum of pomegranate nanoparticles (Fig. 4) showed a peak at 3566 CM^− 1^ indicating O–H Stretching (phenolic compounds, sugars). Also, a peak at 2918 CM^− 1^ indicated C–H Stretching (aliphatic compounds). An additional peak was observed at 1733 CM^− 1^, indicating C= O Stretching (ellagic acid, gallic acid). A peak at 1653 CM^− 1^ corresponds to C = C Stretching (anthocyanins, flavonoids). A peak at 1187 CM^− 1^ indicated C–O Stretching (Glycosidic bonds in pomegranate polyphenols), while a peak at 864 CM^− 1^ indicated glycosidic linkages (aromatic rings in flavonoids).

- X-Ray diffraction (XRD)

**Fig. 5:**
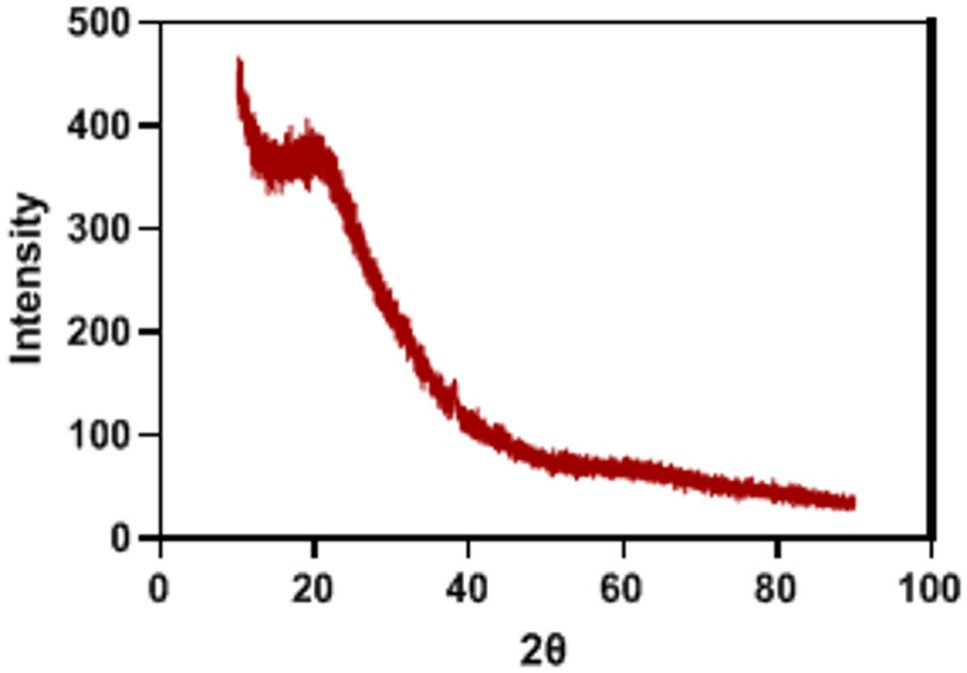
X-Ray diffraction of pomegranate peel extract nanoparticles

The pomegranate-derived material’s X-ray diffraction pattern (Fig. 5) offered vital details on its crystalline constituents, including minerals and crystallized organic molecules. Based on the diffraction pattern, the pomegranate-derived material appears to contain two primary crystalline phases: Calcium Oxalate Phase: Dominant Phase (approximately 75% by intensity), commonly found in plant tissues including pomegranate, Potassium Mineral Phase: Secondary Phase (approximately 25% by intensity), consistent with the high potassium content in pomegranate.

### Characterization of ovarian stem cell-derived exosomes

- Transmission electron microscope (TEM)

**Fig. 6:**
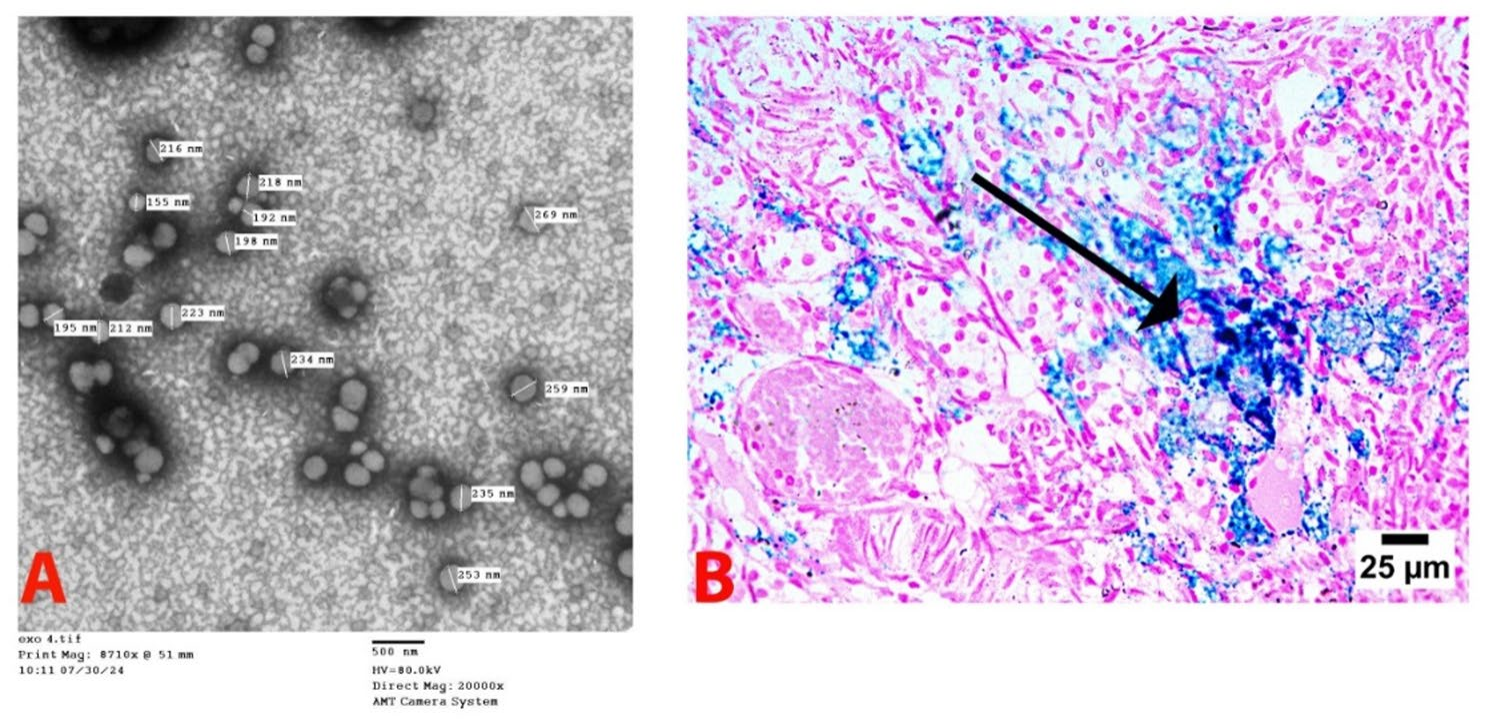
Photograph showing characterization of ovarian stem cell-derived exosomes. **A**: transmission electron microscope images of ovarian stem cells derived exosomes (Mag. 20000×). **B**: arrows indicate iron-labeled exosomes in ovarian tissue (Mallory Prussian blue)

TEM analysis revealed that exosomes had a cup-shaped appearance (Fig. 6A).

- Iron oxide labeling of exosomes

Mallory Prussian blue staining of the ovarian tissue revealed iron oxide nanoparticle granules in the histological analysis. This demonstrated that OSC-derived exosomes migrated to the ovarian tissue (Fig. 6B).

### Hormonal analysis

**Fig. 7:**
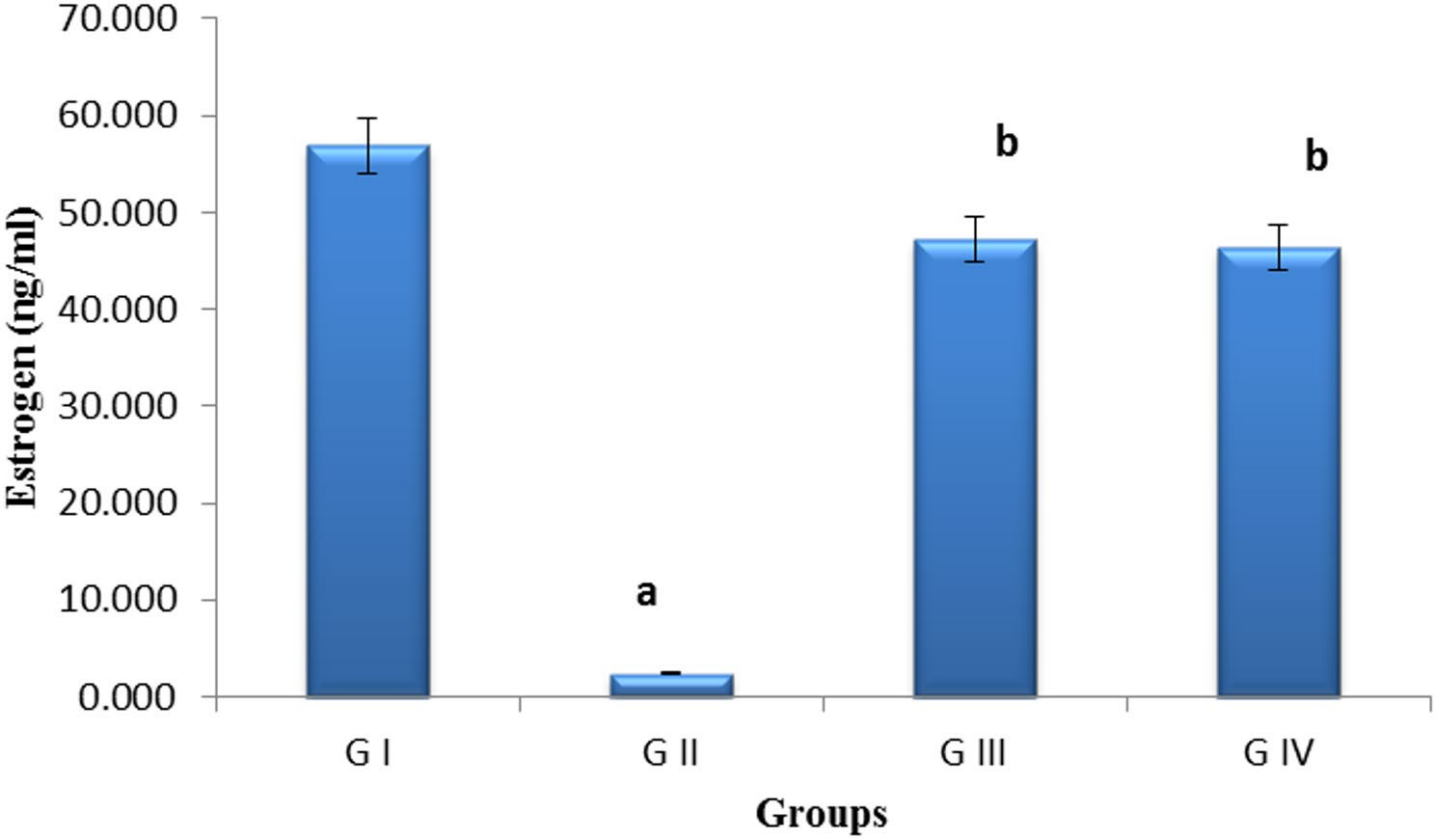
Bar chart showing serum estrogen levels (ng/ml) between groups.** G I** (control negative),** G II** (control positive or CLP group),** G III** (pomegranate nanoparticles treated), GIV (pomegranate nanoparticles + ovarian stem cell-derived exosomes).Data is expressed as means (*n* = 10). (a) indicates significant to control negative group at *P* ≤ 0.05, (b) indicates significant to control positive group at *P* ≤ 0.05

The study found a substantial drop in E2 levels in the cyclophosphamide group (group 2) compared to negative control rats (2.36 ng/ml vs. 56.59 ng/ml, *p* < 0.001). Relative to the positive control, the treatment with pomegranate nanoparticles led to a significant increase in E2 levels (47.18 ng/ml); also, their combination with exosomes led to a noticeable increase in E2 (46.74 ng/ml) when compared to the positive control group (Fig. 7).

**Fig. 8:**
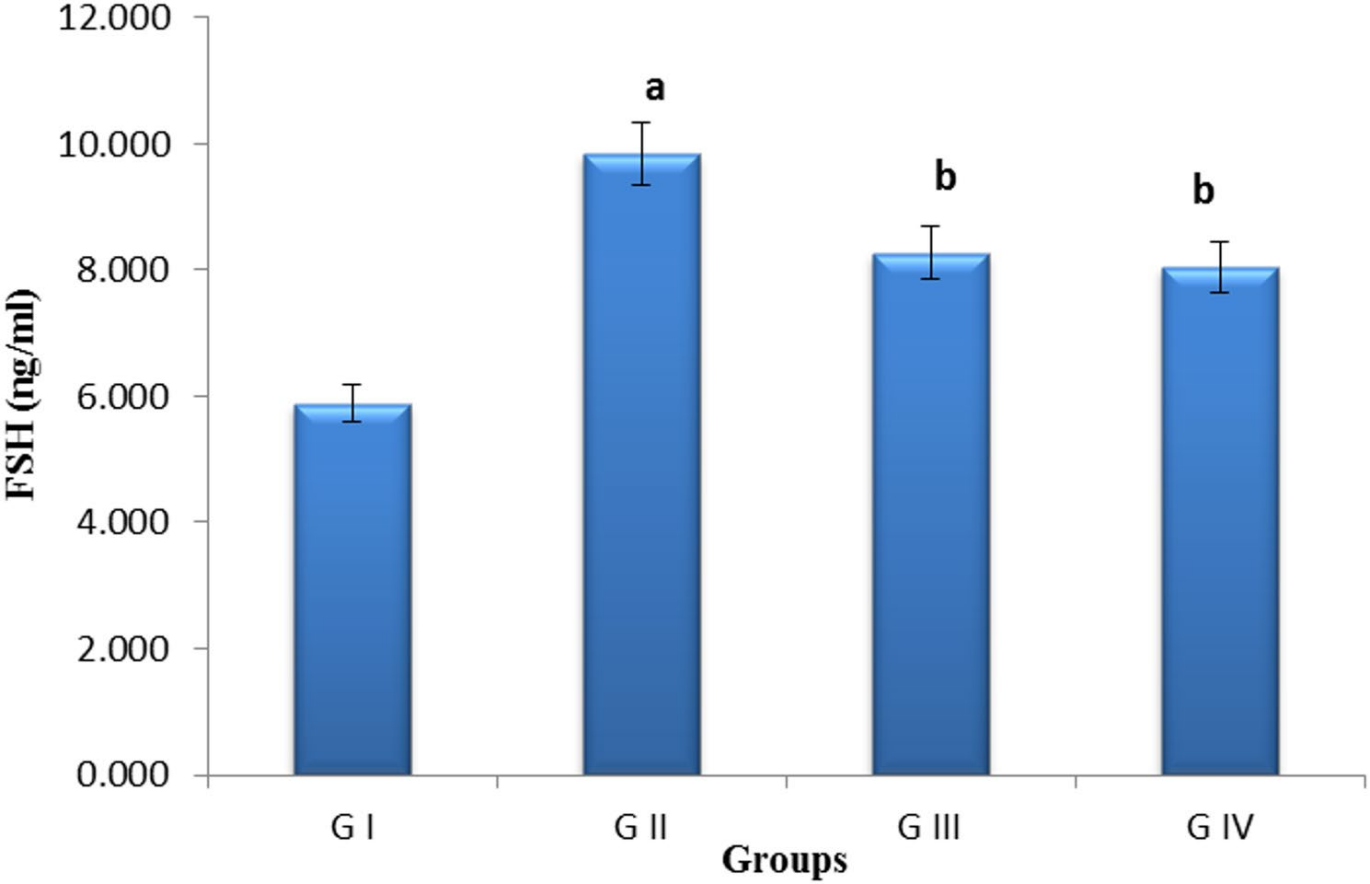
Bar chart showing serum follicle-stimulating hormone (FSH) levels (ng/ml) between groups.** G I** (control negative),** G II** (control positive or CLP group),** G III** (pomegranate nanoparticles treated),** G IV** (pomegranate nanoparticles + ovarian stem cell-derived exosomes). Data are expressed as means. (*n* = 10). (a) indicates significant to control negative group at *P* ≤ 0.05, (b) indicates significant to control positive group at *P* ≤ 0.05

The FSH levels were measured, yielding results of 5.88 ng/ml for the control negative group (group 1), 9.84 ng/ml for the control positive group (group 2), 8.26 ng/ml for the pomegranate-treated group (group 3), and 8.05 ng/ml for the combined treatment group (group 4). Therefore, the administration of pomegranate nanoparticles alone or combined with exosomes resulted in a significant reduction in FSH levels when compared to the control positive group. (Fig. 8**)**.

### Histopathological examination

- Hematoxylin and Eosin stain

**Fig. 9:**
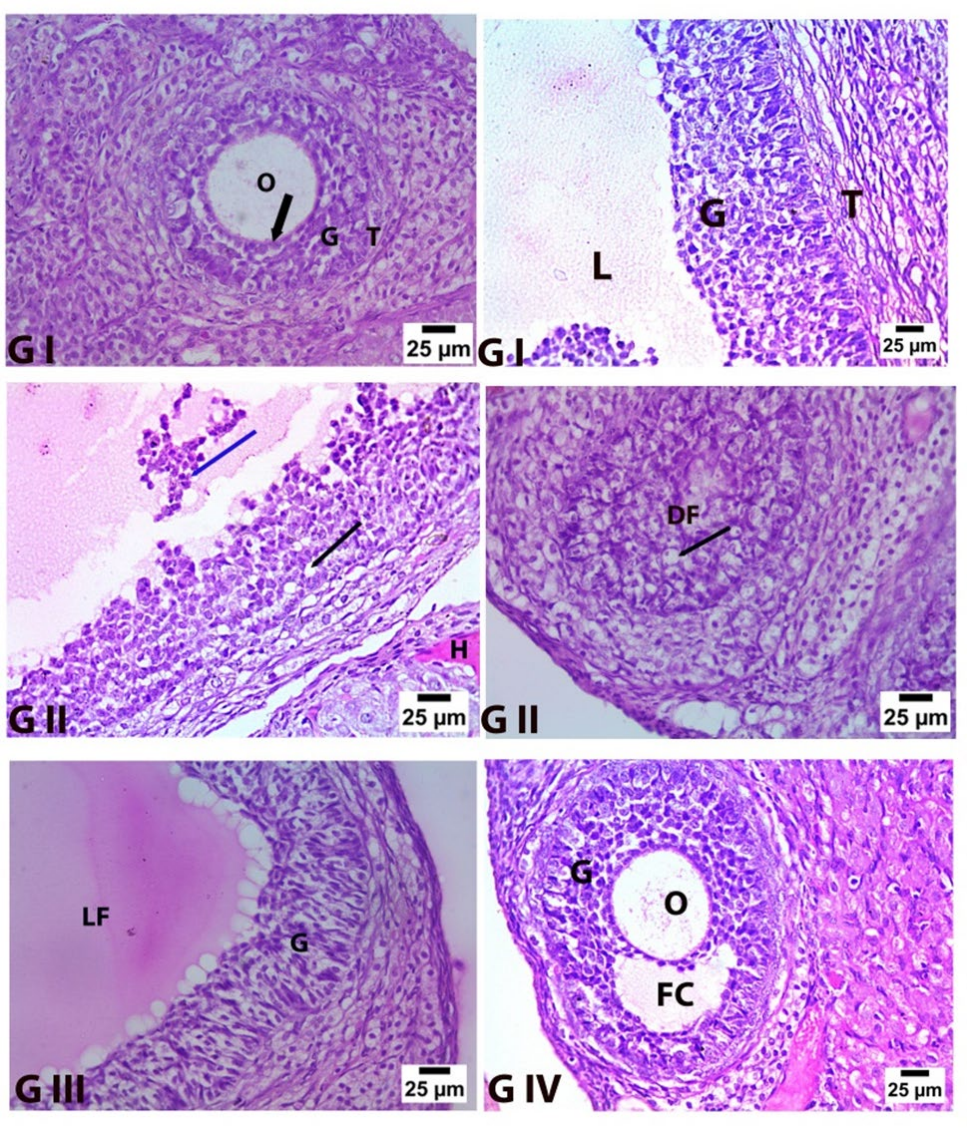
Histopathological evaluation within groups (**G I**) Adult rats’ ovaries in the control negative group showed normal secondary and main follicles containing a normal oocyte (O) surrounded by zona pellucida (arrows). Normal granulosa cell layers (G) and* theca folliculi* (T).* Follicular cavities* (L).** G II** The ovaries of the control positive group exhibited significant granulosa cell vacuolation (black arrows), desquamation of granulosa cells (blue arrows), and hyaline deposits (H).** G III** Ovaries of the pomegranate- treated group: the wall of follicles comprises normally shaped granulosa cells (G) and follicular fluid within the follicular cavity (LF) (Hematoxylin and Eosin stain).** G IV** Ovaries of the pomegranate + exosome co-treated group, revealing a normal oocyte (O), surrounded by a typical layer of granulosa cells (G) with a follicular cavity in between (FC) (H&E, ×400)

Rat ovarian specimens from Group 1 (control negative) have a normal histological structure. The ovary’s surface was composed of a single layer of simple squamous epithelium, a layer of dense connective tissue, and tunica albuginea underneath the epithelium. Growing follicles at different stages of development and corpus luteum were present in the cortex and granulosa cells, which are polyhedral cells that encircle the oocyte. These cells were arranged in a multilayered manner to form a granulosa cell layer Fig. 9(GI). The rats’ ovaries from Group 2 (control positive group) showed substantial degenerative alterations within the cortex; they showed several distinctly deteriorated follicles. These follicles included degenerated oocytes with cytoplasmic vacuoles and pyknotic nuclei, and their granulosa cells had pyknotic nuclei and appeared vacuolated and disordered. In the follicles’ center, a few of these granulosa cells were exfoliated Fig. 9(GII). Ovarian sections from Group 3 (pomegranate nanoparticle-treated group), Fig. 9(GIII), and Group 4 (pomegranate nanoparticles + ovarian stem cell-derived exosomes), Fig. 9(GIV), demonstrated good improvement as shown by reasonably normal ovarian structure. Sections indicated numerous somewhat typical developing ovarian follicles in the ovary’s cortex and mature Graafian follicles. Some ovarian follicles remain in a state of deterioration, with a normal arrangement and appearance of the granulosa cell layer surrounding the oocyte enclosed by the zona pellucida.

**Fig. 10:**
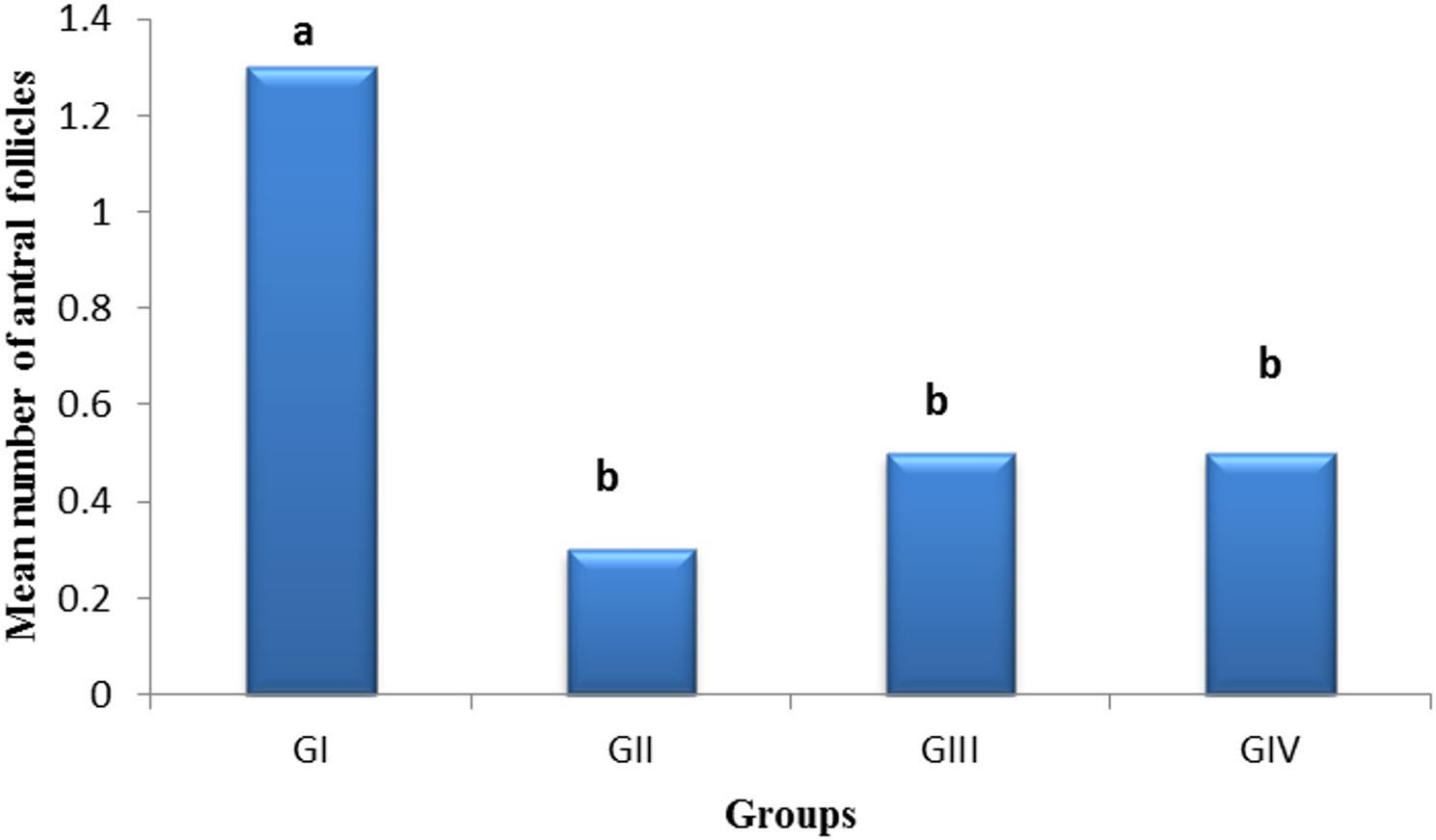
Bar charts represent the mean number of antral follicles in each group.** GI** (control negative),** GII** (control positive or CLP group),** GIII** (pomegranate nanoparticles treated),** GIV** (pomegranate nanoparticles + ovarian stem cell-derived exosomes). Data are expressed as means ± standard error. (*n* = 10). Different subscript letters indicate a significant difference at *P* ≤ 0.05

- Mean number of antral follicles (Fig. 10)

In comparison to the control group, the mean number of antral follicles has significantly decreased (*P* < 0.05) in the group treated with cyclophosphamide. Furthermore, the mean number of antral follicles in the pomegranate-nanoparticles-treated group and in the mixed group (pomegranate peel extract nanoparticles + ovarian stem cell-derived exosomes) has improved when compared to the cyclophosphamide group.

- Proliferating cell nuclear antigen (PCNA) & Caspase 3 immunostaining

**Fig. 11:**
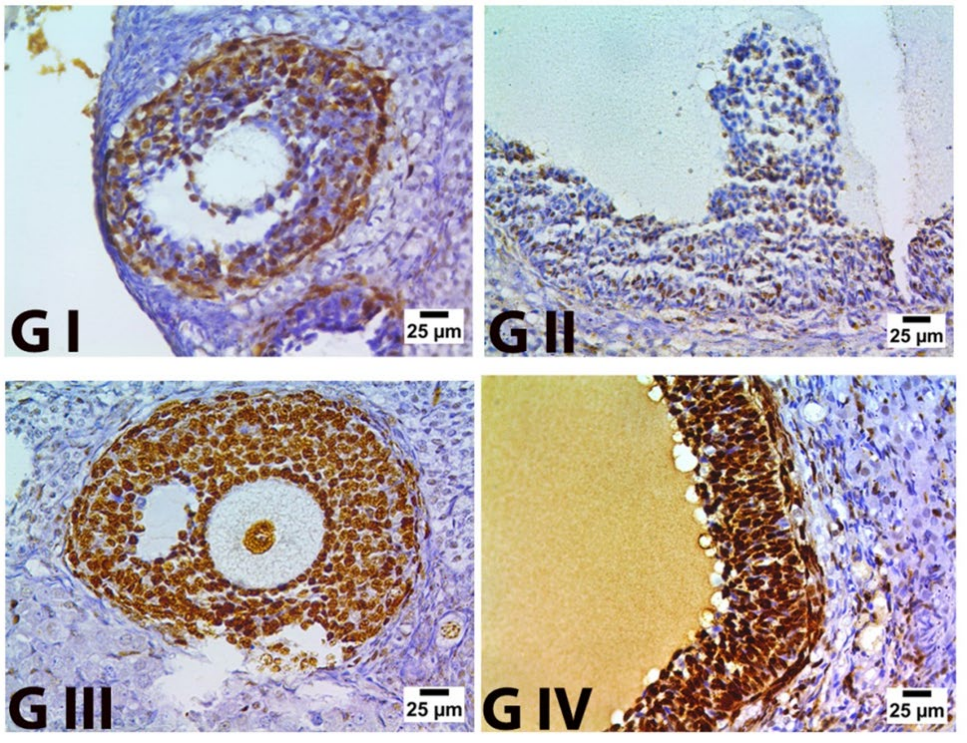
Proliferating cell nuclear antigen (PCNA) immunostaining in ovarian tissue.** G I** (control negative),** G II** (control positive or CLP group),** G III** (pomegranate nanoparticles treated),** G IV** (pomegranate nanoparticles + ovarian stem cell-derived exosomes). Brown pigment indicates expression of PCNA (IHC-Peroxidase DAB) (PCNAX400)

The examination of the control negative group revealed that most granulosa cells and oocyte nuclei exhibited significant PCNA expression, which appeared brown to black in the granulosa cells. Figure 11(GI). Ovarian sections from Group II (the control positive group) showed negative PCNA expression Fig. 11(GII). In Group III (the pomegranate-nanoparticle-treated group), the ovarian sections indicated that most granulosa cell nuclei, which appeared brown to black, exhibited positive PCNA expression. In contrast, some granulosa cell nuclei, which were blue, displayed negative PCNA expression. Figure 11(GIII), on the other hand, sections from group IV revealed strong positive expression of PCNA Fig. 11(GIV).

**Fig. 12:**
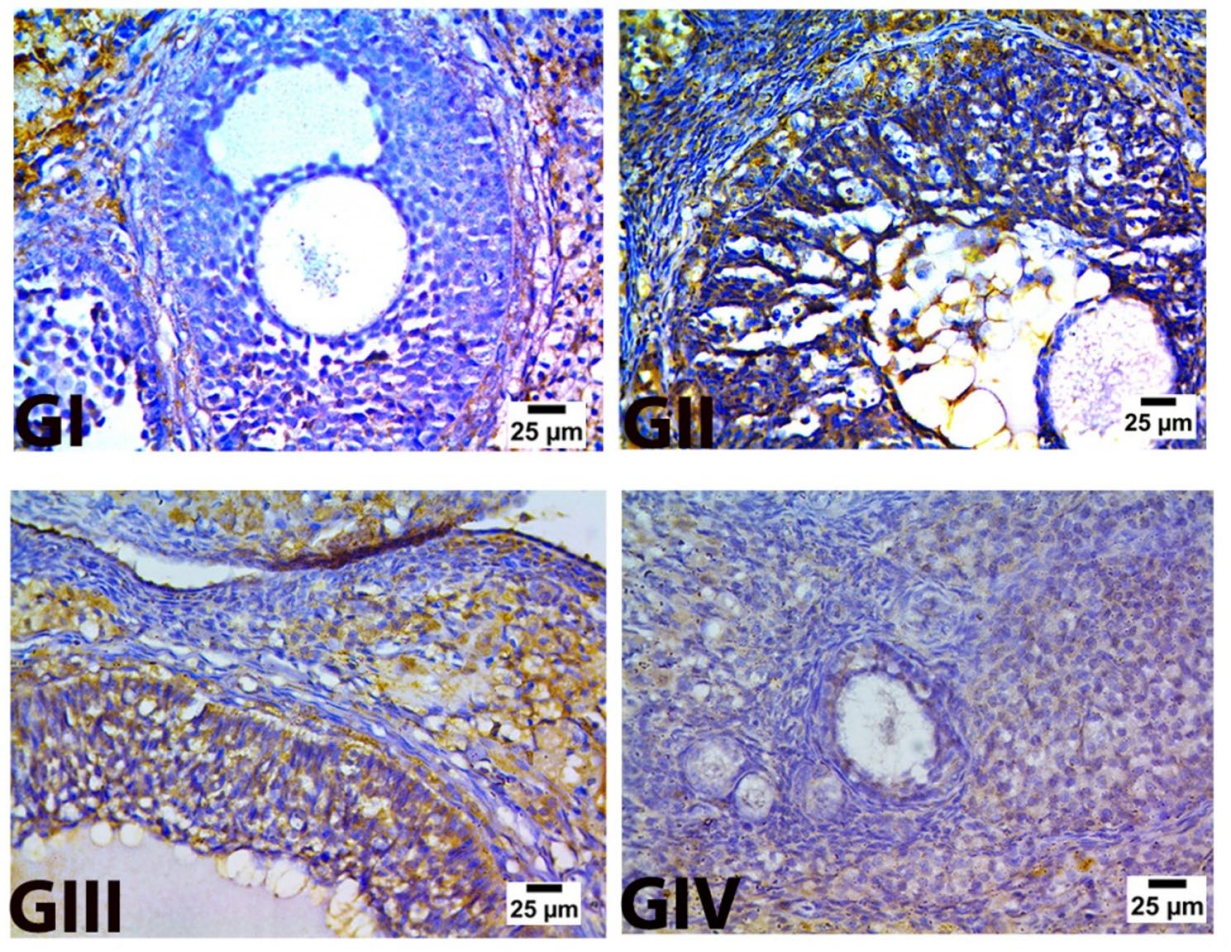
Caspase 3 immunostaining in ovarian tissue.** GI** (control negative),** GII** (control positive or CLP group),** GIII** (pomegranate nanoparticles treated),** GIV** (pomegranate nanoparticles + ovarian stem cell-derived exosomes). Brown pigment indicates expression of caspase 3 (IHC-Peroxidase-DAB). (CaspaseX400)

The cytoplasm of the granulosa, theca cells, and corona radiata of the developing and mature follicles in the control negative specimens showed negative **Caspase 3** expression Fig. 12(G I). While the atretic follicle in the control positive group displayed positive CASP-3 expression in granulosa cells, Fig. 12(GII). Following pomegranate-nanoparticle treatment, specimens displayed either weak or negative **Caspase 3** staining in the granulosa, theca cells, corona radiata, and oocytes of the developing and mature follicles Fig. 12(GIII). Also, samples from group 4 that received the treatment combination showed a very weak reaction of caspase 3, Fig. 12(GIV).

### Oxidative stress parameters

**Fig. 13:**
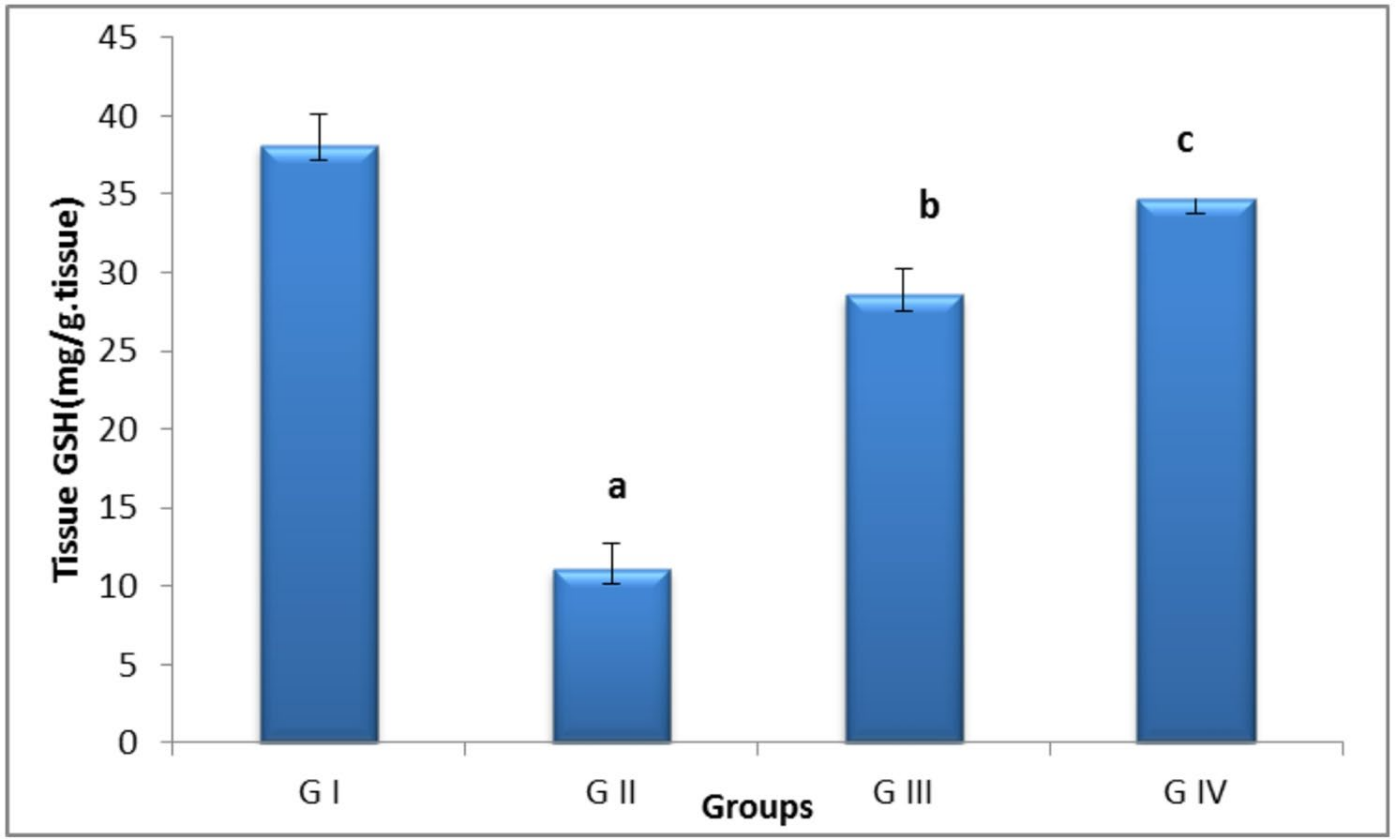
Bar chart showing tissue Glutathione levels (mg/tissue) between groups.**G I** (control negative),** G II** (control positive or CLP group),** G III** (pomegranate nanoparticles treated),** G IV** (pomegranate nanoparticles + ovarian stem cell-derived exosomes). Data are expressed as means ± standard error. (*n* = 10). Different subscript letters indicate significant difference at *P* ≤ 0.05

**Fig. 14:**
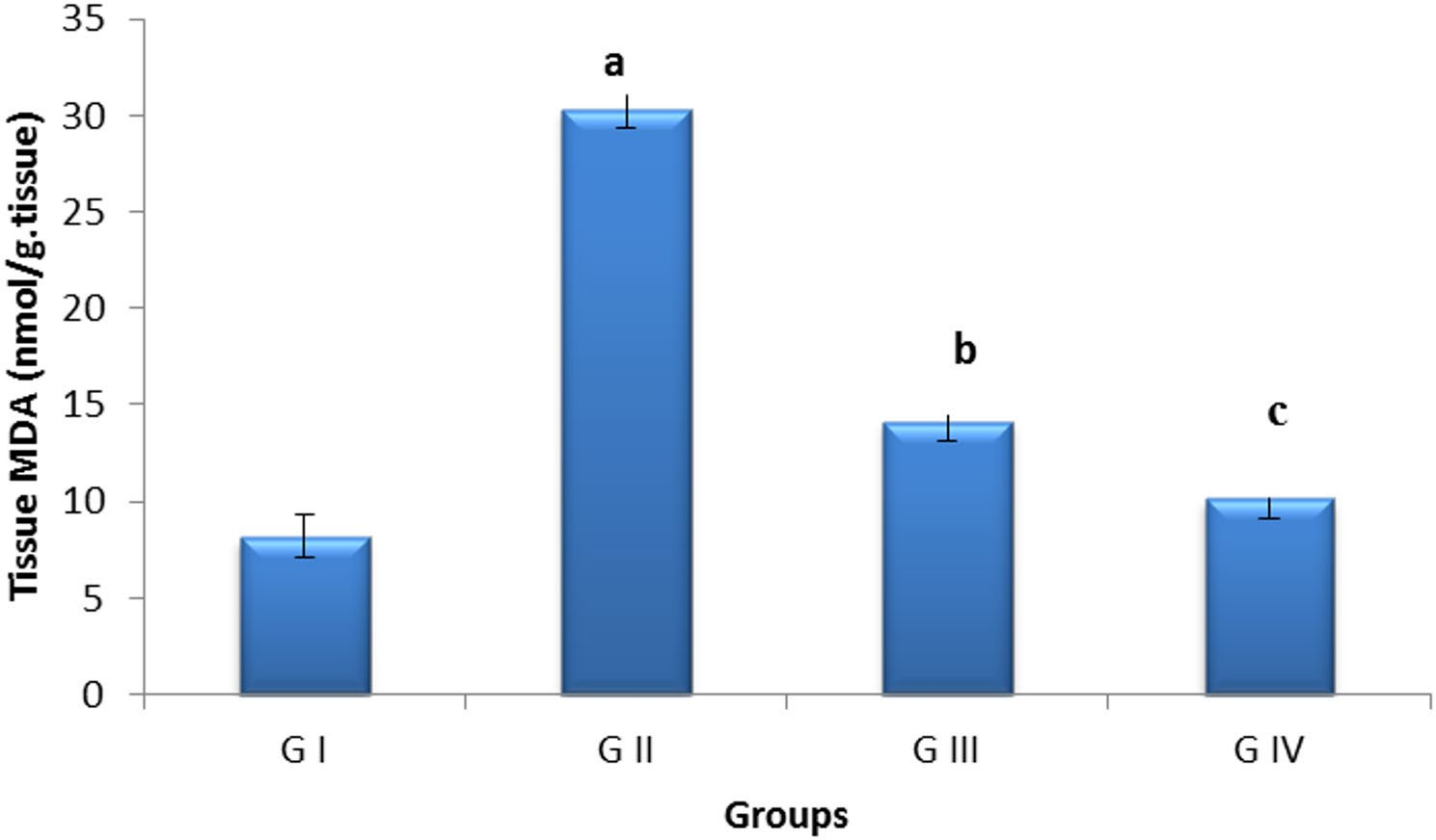
Bar chart showing tissue Malondialdehyde levels (nmol/g tissue).** G I** (control negative),** G II** (control positive or CLP group),** G III** (pomegranate nanoparticles treated),** G IV** (pomegranate nanoparticles + ovarian stem cell-derived exosomes).Data are expressed as means ± standard error. (*n* = 10). (Different subscript letters indicate significant difference at *P* ≤ 0.05

Rats in the control positive group had higher malondialdehyde (MDA) levels and decreased glutathione (GSH) levels in ovarian tissue relative to the control negative group. The pomegranate nanoparticles mitigated cyclophosphamide-induced oxidative damage to the ovary by elevating ovarian GSH and reducing MDA levels in the pomegranate co-treated group; however, the combined treatment had better results than nanoparticles alone, as shown by higher GSH levels and lower MDA. (Figs. 13 and 14).

### Gene expression of AMH, CYP 19a1, and FSHR

**Fig. 15:**
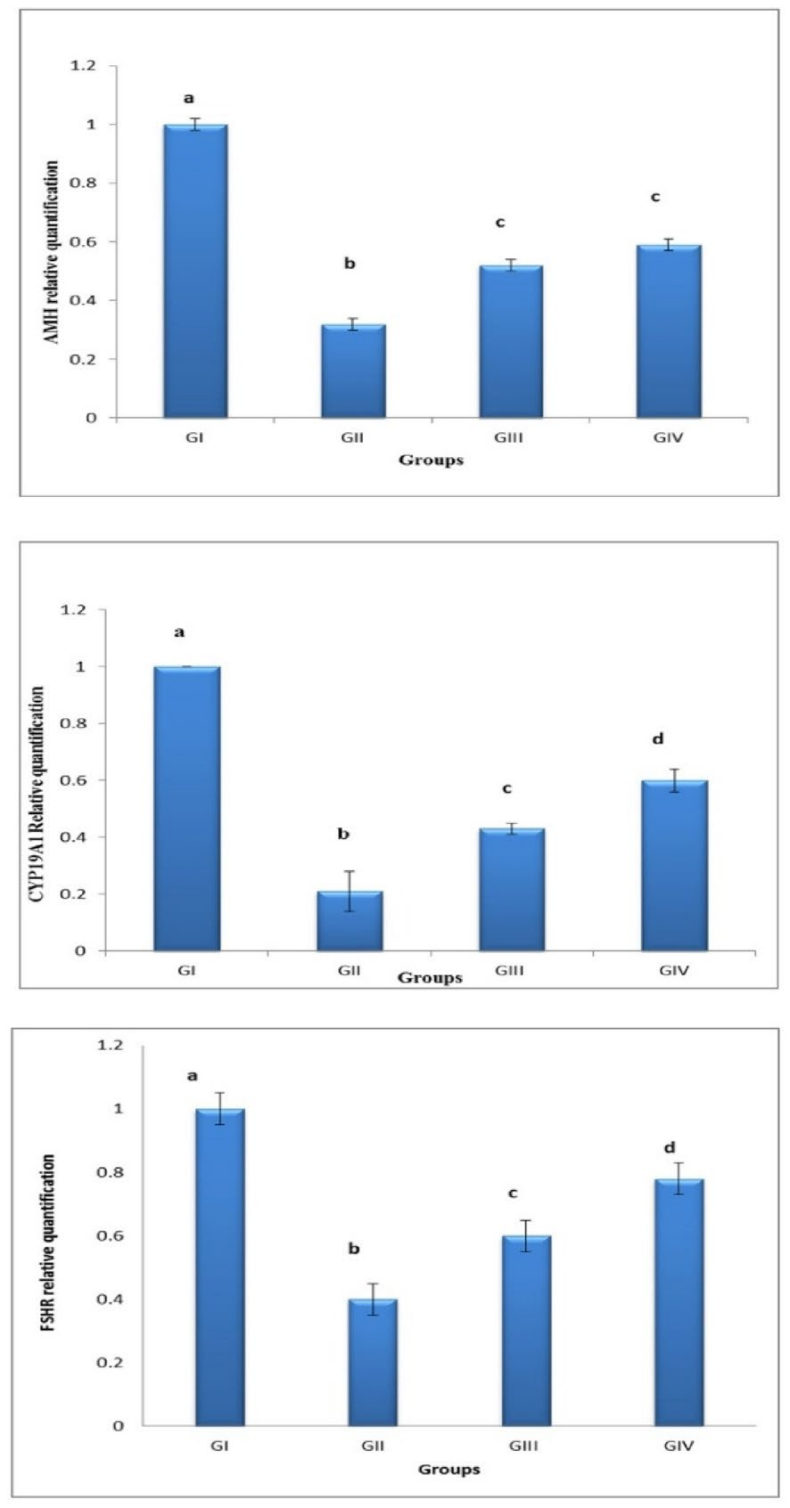
Bar chart showing expression patterns of AMH, CYP19a1, and FSHR genes between the different groups.**GI** (control negative),** GII** (control positive or CLP group),** GIII** (pomegranate nanoparticles treated), GIV (pomegranate nanoparticles + ovarian stem cell-derived exosomes). Data is expressed as means ± standard error. (*n* = 10). Different subscript letters indicate a significant difference at *(*p* ≤ 0.05)

Compared to the cyclophosphamide group, the control group had greater levels of AMH, CYP19a1, and FSHR expression. Additionally, the group treated with pomegranate nanoparticles had significantly higher levels of AMH, CYP19a1, and FSHR gene expression than the group treated with cyclophosphamide. Also, the combined treatment group had a superior effect compared to pomegranate only; this was evidenced by the higher levels of gene expression. These findings suggest that pomegranate nanoparticle treatment, either alone or combined with OSCs exosomes, improved the expression levels of genes specific to ovarian granulosa cells (Fig. 15).

## Discussion

This study examined the potential therapeutic effect of pomegranate peel extract nanoparticles, independently and in conjunction with OSC-derived exosomes, on cyclophosphamide-induced premature ovarian insufficiency by evaluating ovarian structure, function, oxidative stress markers, and gene expression.

The specific mechanism through which CLP causes this damage is unknown, but it is either caused by the acceleration of the natural aging process of the follicles, which in turn causes loss of follicles and reduction in the process of folliculogenesis [33–35], or the release of active metabolites such as phosphoramide mustard and acrolein. These metabolites can attach to DNA, which can result in cell death and interruption of DNA synthesis [35, 36]. Also, CLP metabolites generate reactive oxygen species (ROS), which in turn suppress the ovary’s antioxidant defense system [34, 37, 38].

Pomegranate possesses a diverse array of pharmacological activities, including antibacterial, antifungal, antitumor, anti-inflammatory, and anti-diabetic effects [39–41]. Also, pomegranate has a significant antioxidant effect due to its content of polyphenols, including hydrolyzable tannins and flavonoids [42–44].

The overall FTIR profile indicates that the pomegranate-derived material contains a rich mixture of polyphenols (particularly ellagitannins), anthocyanins, flavonoids, and fatty acids, consistent with the known composition of pomegranate extracts. This was in line with Gullon et al. [45], Abid et al. [46], Vinay et al. [47].

XRD results offered details on the crystalline mineral components. When the results of XRD and FTIR are combined, they point to a substance that contains both organic bioactive chemicals and mineral nutrients (potassium, calcium). The bioavailability and bioactivity of pomegranate antioxidants may be impacted by combinations that the calcium oxalate crystals may form with the polyphenolic chemicals. Also, the antioxidant and anti-inflammatory properties of pomegranates may be strengthened by this combination. These results were supported by Gullon et al. [45, 47], Hasnaoui et al. [48].

Exosomes can initiate the processes of regeneration and repair to restore tissue homeostasis and vital biological functions. This is done through signaling molecules that act in a paracrine manner to target cells, resulting in the regulation of specific molecular cascades [49]. Exosomes can improve tissue reactions to illness and injury. Their clinical uses for POI are improved by these features. As a result, this cell-free regenerative medication offers POI a novel, low-risk treatment. Several studies reported the use of exosomes in the treatment of several diseases, such as Fan et al. [50] in central nervous system disorders, Han et al. [51] in pancreatic disease, Malekpour et al. [52] in orthopedic diseases and Wu et al. [53] in testicular injury caused by Cisplatin.

In our study, exposure to cyclophosphamide led to a significant drop in ovarian function and periodicity of the cycle, which was clear as only 10% of rats in the POI group experienced a normal estrus cycle; the rest suffered from either no periodicity or prolonged diestrus and a short estrus phase in comparison to the control. These results were similar to the findings of Ling et al. [54], Li et al. [55].

The pomegranate nanoparticle treatment results in a significant improvement in ovarian architecture characterized by the normal organization of granulosa cells, the absence of vacuolation or desquamation, and the existence of numerous follicles at various developmental stages, including mature Graafian follicles. This benefit resulted directly from the anti-apoptotic and antioxidant properties of pomegranate, which mitigated the oxidative stress induced by CLP metabolites, as elevated cellular glutathione and glutathione s-transferase levels result in resistance to cyclophosphamide in cell lines [56, 57]. This finding aligns with Almuhayawi et al. [13] in the treatment of Alzheimer’s.

Exosome injection with pomegranates resulted in better protection through controlling immune cell responses and preventing ovarian granulosa cells’ (GCs’) apoptosis, also with its potent anti-inflammatory and antioxidant effects. This was similar to Li et al. [58], Park et al. [59].

The anterior pituitary secretes FSH, which stimulates and facilitates the development of ovarian follicles. FSH receptors are primarily expressed on GCs in follicles. Theca cells and GCs in follicles release E2; pituitary FSH secretion is negatively impacted by a declining E2 level in the hypothalamus-pituitary-ovarian axis [54, 60].

A POI model was created in this study using CLP, which can cause GC apoptosis, follicular loss, vascular damage, tissue fibrosis, and ovarian dysfunction. Our findings demonstrated that CLP caused a reduction in the quantity of antral follicles in the ovaries of rats in the POI group. As a result, CLP led to a drop in serum levels of E2. The serum FSH level rose as a result of the negative feedback caused by the lower E2 level, which was similar to the findings of Ling et al. [54], Li et al. [55], Fouad et al. [61], Vural et al. [62]. Treatment with pomegranate nanoparticles, either alone or in conjunction with exosomes, significantly increased E2 levels relative to the control positive group. This was a direct consequence of the antioxidant effect of pomegranate and the antiapoptotic ability of exosomes as evidenced by decreased apoptosis of granulosa cells, consequently diminishing FSH through a negative feedback mechanism. This was in line with Park et al. [59], Nazdikbin et al. [63].

The examination of the control positive group’s ovaries by H&E demonstrated a considerable depletion of follicles, accompanied by notable shrinkage and distortion, as well as the presence of many atretic follicles. Additionally, several follicles exhibited disorganization because of the death of granulosa cells and their detachment from the base of the follicles. This finding was analogous to Mohamed et al. [28], Li et al. [55], Nazdikbin et al. [63], Besikcioglu et al. [64]. Pomegranates also alleviated some of the oxidative stress caused by CLP metabolites also Exosomes infusion with pomegranate nanoparticles resulted in less granulosa cell apoptosis, hence, less follicular atresia and more morphologically sound follicles than in the POI control group; similar results were reported by Yamchi et al. [26], Huang et al. [65].

The current study’s immunohistochemical analysis of pomegranate co-treated ovarian sections stained with PCNA revealed a mild positive reaction in the majority of granulosa cells in the form of dark brown nucleus pigmentation, indicating greater cell proliferation and regeneration when compared to the control positive group, which had negative expression, as CLP exposure resulted in apoptosis of granulosa cells, which is compatible with Pu et al. [15], Mohamed et al. [28]. Caspase 3, referred to as the execution caspase, initiates apoptosis upon activation. Our findings demonstrated a pronounced expression of Caspase-3 in the granulosa cells of the positive control group, indicating its substantial function in the regulation of granulosa cell death. This finding was supported by Liu et al. [66] in mice. On the other hand, sections from the ovaries of the pomegranate nanoparticle-treated group and the combined treated group had very mild or negative expression of CASP-3 versus strong PCNA expression, which could contribute to the recovery of fertility and ovarian function following treatment, similar to results reported by Zhang et al. [34] in mice.

The ovarian granulosa cell-specific genes AMH, FSHR, and CYP19a1 were evaluated for expression levels using real-time PCR. The results showed that the control group’s levels of AMH, CYP19A1, and FSHR gene expression were significantly higher than those of the CLP group, which was a direct effect of cyclophosphamide on theca cells and granulosa cells, hence folliculogenesis, and that the pomegranate-treated group’s levels of AMH, FSHR, and CYP19A1 expression were higher than those of the CLP group. This was in line with Manshadi et al. [67], Vural et al. [62] The combined treatment group had the best results in elevating expression levels of granulosa-specific genes, similar to results reported in Park et al. [59] These findings suggest that treatment with pomegranate nanoparticles, either alone or with OSCS exosomes, had a beneficial impact on the expression levels of ovarian granulosa cell-specific genes.

Cyclophosphamide’s ROS can cause oxidative damage, which can harm the ovaries. In the present investigation, cyclophosphamide caused detrimental effects on the antioxidant defense mechanisms, as demonstrated by a notable rise in MDA and a decline in GSH. Free oxygen species can cause disruption of cadherin/catenin complexes and oxidative phosphorylation of cell membranes, which compromises the integrity of the junctional complex and initiates oxidative damage and cell death, in agreement with Zhang et al. [34], Lopez et al. [68], Ismail et al. [69]. Treatment with pomegranate nanoparticles ameliorated the oxidative stress level caused by cyclophosphamide metabolites, as indicated by the significant rise in glutathione levels and reduction in MDA levels; this is attributed to the potent antioxidant effect of pomegranate and its content of polyphenol. Furthermore, the synergy between pomegranate and exosomes resulted in better protection of the ovarian tissue against oxidative damage caused by CLP, as evidenced by the higher levels of GSH and lower levels of MDA in comparison with pomegranates alone [42, 43, 70].

The study investigates the protective impact of pomegranate (*Punica granatum*) peel extract nanoparticles, independently and in conjunction with exosomes generated from OSCs, against chemotherapy-induced POI by several techniques. Nonetheless, additional study is required to investigate other underlying processes for this role, as well as experiments including different species.

In addition to the experimental groups presented in the current study, a group treated with exosomes only (ovarian stem cells–derived exosome group) was included as part of a broader experimental design conducted in a subsequent, yet unpublished work. This group received exosomes only and was not incorporated in the present manuscript to maintain experimental consistency. The exosome-only group demonstrated a noticeable elevation in serum estradiol levels, accompanied by a reduction in follicle-stimulating hormone (FSH), indicating a partial recovery of ovarian endocrine function. This was similar to Pu et al. [15]. Moreover, tissue biochemical analyses showed a higher glutathione concentration and a lower malondialdehyde (MDA) level, reflecting improved oxidative balance, which was in line with Ren, He [71]. At the molecular level, gene expression analysis revealed upregulation of AMH, CYP19, and FSHR consistent with the hormonal findings and suggesting enhanced follicular responsiveness. Similar results were reported by Park et al. [59], Huang et al. [72]. Although these data remain unpublished, they complement the current findings and further support the potential role of exosome-based therapy in promoting ovarian function restoration. Overall, the exosome-treated group exhibited a statistically significant improvement across all evaluated parameters compared with the negative control group. This trend was generally comparable to the responses observed in the treatment groups presented in the current study, with the most pronounced effect being achieved in the combination group that received both the nano-formulation and exosomes.

## Conclusion

Pomegranate (*Punica granatum*) peel extract nanoparticles markedly enhanced ovarian insufficiency in rats induced by chemotherapy owing to the robust antioxidant and antiapoptotic properties of pomegranates. Also, the combination of pomegranates and exosomes resulted in yet better results than pomegranates alone due to the regeneration ability and the antioxidant effect of exosomes. However, this study has certain limitations, being performed in a rat model, which may not fully mimic the normal human ovarian physiology. We recommend future studies evaluating more treatment parameters, such as (fertility index, pregnancy ratio, and live/dead offspring ratio), and advance to some human clinical trials.

## Data Availability

All data generated or analyzed during this study are included in this published article.

## Abbreviations

CLP: Cyclophosphamide
POI: Premature ovarian insufficiency
OSCs: Ovarian stem cells
PBS: Phosphate-buffered saline
DMEM: Dulbecco’s modified eagle medium
FSH: Follicle-stimulating hormone
E2: Estrogen
PCNA: Proliferating cell nuclear antigen
GSH: Glutathione
MDA: Malondialdehyde
ROS: Reactive oxygen species
CYP19a1: Cytochrome P450 Family 19 Subfamily A Member 1
AMH: Anti-Müllerian hormone
FSHR: Follicle-stimulating hormone receptor
XRD: X-Ray diffraction
ATR: Attenuated total reflection
FTIR: Fourier transform infrared spectroscopy
CCG: Carbon-coated copper grids
TEM: Transmission electron microscope
